# A review on B-type natriuretic peptide monitoring: assays and biosensors

**DOI:** 10.1007/s10741-016-9544-9

**Published:** 2016-03-15

**Authors:** Rita Maalouf, Steven Bailey

**Affiliations:** 1 Department of Sciences, Faculty of Natural and Applied Sciences, Notre Dame University Louaize, P.O. Box 72, Zouk Mikael, Zouk Mosbeh, Lebanon; 2 Division of Cardiology, Department of Medicine, University of Texas Health Science Center at San Antonio, 7703 Floyd Curl Dr., San Antonio, TX 78229-3900 USA

**Keywords:** Brain natriuretic peptide, Biomarker, Heart failure, Implantable biosensor

## Abstract

Since its discovery in 1988, B-type natriuretic peptide (BNP) has been recognized as a powerful cardiovascular biomarker for a number of disease states, specifically heart failure. Concurrent with such a discovery, much effort has been allocated to the precise monitoring of physiological BNP levels. Thus, it can be used to guide the therapy of heart failure and determine the patient’s stage of disease. Thus, we discuss in this article BNP as a potent biomarker. Subsequently, we will review the progress of biosensing devices as they could be applied to monitor BNP levels as assays, benchtop biosensors and implantable biosensors. The analytical characteristics of commercially available BNP assays are presented. Still emerging as a field, we define four obstacles that present opportunity for the future development of implantable biosensor: foreign body response, sensor renewability, sensitivity and selectivity.

## Introduction

A recent report from the American Heart Association estimated that 85.6 million Americans over the age of 20 have one or more types of cardiovascular disease (CVD). Of these, 5.7 million are estimated to have heart failure (HF). One in nine deaths has HF mentioned on the death certificate. This was expected to cost billions of US dollars in medical services, medications and lost productivity; HF represents a significant drain on health resources both in the US and in many other nations. Projections show that by 2030, the prevalence and the total cost of HF will increase 46 and 127 %, respectively [1].

While an early diagnosis may help control the condition and prevent rapid decline, it would obviously be preferred to identify those patients at high risk of HF and offer preemptive specific and aggressive treatment before its onset. Thus, began the search for a window into HF and a metric to quantify the condition.

### Why biomarkers?

Biomarkers are biological analytes detectable in the blood stream under specific physiological conditions [2]. As such, biomarkers are a cost-effective, precise way of peering through a patient’s physiological window and differentiating disease states in complex patients [3]. In particular, cardiovascular biomarkers promise to offer such a window into HF. Though controversy exists regarding the existence of an ideal cardiovascular biomarker to be considered useful in monitoring HF [2], an ultimate biomarker would be highly sensitive and specific for HF, be released quickly for early diagnosis, be persistent for a reasonable amount of time to allow for a diagnostic window and be quantified accurately and economically in a manner that would reflect changes in both the patient’s clinical status and prognosis [4, 5]. The biomarker may be a by-product of the disease state and may also directly participate in its pathogenesis or modulation [6]. Among cardiovascular biomarkers, brain natriuretic peptide and N**-**terminal pro-B-type natriuretic peptide (NT-pro-BNP) have emerged as powerful diagnostic tools for acute HF and as screening tools for detecting left ventricular systolic and diastolic dysfunction [7–10]. Several clinical trials have also evaluated the benefit of monitoring BNP and NT-pro-BNP concentrations to guide the therapy and the management of chronic heart failure [11–13]. Some studies reported encouraging trends [14–17], and others showed benefits in subgroups of participants [18–20], while other trials showed no advantages of peptide-guided compared to clinically guided therapy [21–25]. On the other hand, a recent meta-analysis, conducted by Savarese et al. [26] on 2686 patients from the previously stated 12 trials, showed that natriuretic peptide-guided therapy is associated with outcome benefits.

### Scope of this review

This review aims to survey the fields of BNP research and analyte detection techniques, specifically implantable BNP biosensors. Volumes have been dedicated both to the elucidation of BNP and biosensor technology. In this review, detail will be provided for the purpose of allowing the reader to grasp the skeleton concepts, thereby providing a foundational appreciation for BNP as a biomarker as well as for the necessity of an effective instrument to measure BNP. Our hope is to accomplish this by providing an overview of current and past BNP research without becoming encumbered by minutia. Should the reader like to engage the overwhelming amount of the BNP literature, we have attempted to provide ample references that will serve as a springboard for further research.

The field of biosensors is vast and constantly progressing; a complete account is beyond the scope of this review. After a brief introduction of assays and what we will consider benchtop biosensors, our gaze will fall more specifically upon implantable biosensors. While examples of implantable biosensors will be mentioned to provide something of a disciplinary context and to provide an appreciation of the challenges experienced in implantable biosensor development, we will focus discussion on those challenges and potential for growth as it relates more specifically to BNP implantable biosensors.

## BNP

### Discovery and structure of BNP

Originally named porcine brain natriuretic peptide (pBNP) after the porcine brain from which it was first derived, pBNP is a 26 amino acid chain that shares considerable sequence similarity with alpha human atrial natriuretic peptide (α-hANP) [27]. Another form with an additional six residues at the N-terminus, BNP_1-32_ [28] and a larger precursor form, 12 kDa, have been found, respectively, in the pig brain and heart [29]. It soon became clear that the main source of this natriuretic peptide was the heart rather than the brain [30, 31].

### Stimulus, production and processing

BNP is produced predominantly in the left ventricular myocardium in response to myocyte distension caused by ventricular volume expansion or pressure overload [32–35]. Though somewhat unclear, it seems that BNP is regulated by post-translational processing; therefore, its gene product can increase very rapidly in response to stimulus [33, 36]. This is significant because ANP, which shares a similar stimulus, is regulated at the level of release from storage granules and therefore does not increase as rapidly or in as great a magnitude [33, 36, 37].

When threshold is reached, BNP is produced as a 134 amino acid pre-pro-BNP peptide that is cleaved soon after secretion to yield the 108 amino acid pro-BNP. Further cleavage produces the biologically active 32 amino acid BNP peptide (BNP_1-32_) which contains a 17 amino acid ring closed by a disulfide bond and also the linear 76 amino acid N-terminal peptide (NT-pro-BNP_1-76_) [33]. The biologically active BNP_1-32_, the pro-BNP and the NT-pro-BNP_1-76_ all can be measured in the plasma, as will be described below [36, 37].

### Mechanism of action

Natriuretic peptides bind to three kinds of membrane bound natriuretic peptide receptors (NPR): NPR-A, NPR-B and NPR-C. These receptors and their respective activations are described in some detail by Vanderheyden et al. [36] and will not be delineated here. However, it is important to note that BNP_1-32_ mediates its biological actions by binding to NPR-A, which is abundant in the vascular endothelium system and is also present in the brain, the kidney and the adrenal glands [38–40]. BNP has been shown to be a counter-regulatory molecule for the renin–angiotensin–aldosterone system, to improve glomerular filtration rate and filtration fraction and to have diuretic, natriuretic and vasodilatory effects [41]. Thus, BNP dilates blood vessels, lowering vascular resistance while facilitating higher stroke volume, and also causes increased sodium secretion from the kidneys, increasing urine production while decreasing blood volume and therefore blood pressure.

### Degradation and elimination

The degradation and elimination mechanisms of BNP are only partly understood and are somewhat surrounded by controversy [42–45]. Of course, these must be further researched whether BNP is to be used as a predictable and precise biomarker of physiological condition; if it is to be a window into an internal physiology, we must know how long the window opens, why it opens, what causes the window to close and how clearly we can see through. Information will be presented here to give an appreciation of the complexity of tracking the moving target that is BNP. At present, research has demonstrated that the combined clearance of BNP by NPR-C, the cleavage and inactivation by neutral endopeptidase (NEP) and the clearance by normal glomerular filtration contribute to an estimated half-life of 20 min compared to 90–120 min for NT-pro-BNP which is only eliminated by renal filtration [36, 44, 46–48]. In addition to the NEP, BNP is also cleared through proteolysis by other peptidases including serine proteases, peptidyl arginine aldehyde proteases and kallikrein-like proteases [48].

A complication to measuring BNP concentrations (or any degradable biomarker, for that matter) is its sensitivity to degradation. Proteolytic cleavage of BNP’s highly sensitive N-terminal residues may begin within circulation or immediately after blood collection making precise measurements of physiological levels difficult to achieve. Chromatographic fractionation and analysis of plasma from heart failure patients collected in siliconized tubes to avoid contact activation of kallikreins have suggested that prior to any in vitro activation of proteases, there is a natural in situ activity that results in in vivo cleavage of the two amino acids at the N-terminus (Ser-Pro) [49]. The dipeptidyl peptidase IV, a serine protease that cleaves the N-terminal dipeptide after a proline amino acid, was proved to be responsible of this cleavage [42].

C-terminal proteolysis can occur in vivo and/or in vitro by activation of the kallikrein contact-activated system [50]. It has been reported that both the disulfide bond-mediated ring and the carboxyl terminal structure are stable in blood samples unless blood coagulation factors are activated, which often occurs when blood comes into contact with foreign surfaces, such as a glass test tube [51]. This type of degradation can be inhibited by using EDTA and siliconized glass tubes for biofluid collection and by the addition of kallikrein and serine protease inhibitors [49, 52–54].

Despite the important physiological role played by BNP as a natural hormone and its importance as a diagnostic analyte, little is known of the structure of circulating BNP and of its immunoreactivity, both of which are essential for its quantification. Various groups have found that BNP exists in plasma as two major forms: low molecular weight and high molecular weight corresponding to BNP_1-32_ (~3–4 kDa) and to pro-BNP_1-108_ (~30–40 kDa), respectively. Reversed-phase HPLC of extract from low molecular weight fraction of gel-filtration HPLC has shown that BNP_1-32_ is formed during blood circulation and consists of digested pro-BNP_1-108_. Little immunoreactivity was observed for the BNP fragments: BNP_1-32_, BNP_3-32_, BNP_4-32_ and BNP_10-32_ [45]. The high molecular weight which is about three or four times as large as that of pro-BNP (~12 kDa) and was reported by some authors to be the oligomerization product of pro-BNP which tend to form trimers or tetramers due to a leucine zipper motif in the pro-region [49, 53, 54]. Schellenberger et al. [55] have demonstrated that BNP precursor is an O-linked glycoprotein and that the high molecular weight is likely due to the glycosylation [55, 56]. It has been published also that pro-BNP is the predominant form displaying BNP immunoreactivity in patients with heart failure [56, 57].

### As a marker for heart failure

BNP has emerged as a powerful sensitive and specific diagnostic and prognostic tool for the onset of acute heart failure and as a screening tool for detecting left ventricular systolic and diastolic function [8]. HF is characterized by a decrease in stroke volume, leading to insufficient cardiac output to meet the body’s needs. Concomitant with this decrease in stroke volume is an increase in right atrial pressure or filling pressure. This high filling pressure stretches the walls of the heart, causing BNP release as a kind of biological call for help, as described above, decreasing blood volume and therefore blood pressure. Though BNP may not be an appropriate biomarker for all cardiovascular conditions, it has been sufficiently demonstrated to be specific for numerous disease states.

BNP can serve as a biological marker in more than the stereotypical adult patient with HF. Recent studies have demonstrated that BNP is significantly higher in neonates with congenital heart disorders, such as hemodynamically significant left to right shunts [2, 58]. Pregnant women with severe preeclampsia, the most common pregnancy disorder resulting in significant maternal and neonatal morbidity and mortality, have also been observed to have elevated BNP level [2].

As a diagnostic tool, BNP can help narrow down a differential diagnosis; i.e., it can be used to differentiate dyspnea due to HF (high BNP levels) from dyspnea due to other causes (normal BNP levels) [59, 60]. Early diagnosis is critical, specifically in the elderly population, where misdiagnosis of HF can easily and rapidly lead to morbidity and mortality [61]. BNP levels also predict sudden cardiac death and ventricular arrhythmias [60].

Because BNP can also be used to measure the severity or progress of HF, it can also be used to monitor treatment efficacy in acute HF patients, wherein levels would be expected to decrease, albeit not original values [61–63]. Current studies are underway to further test the efficacy of HF treatment guided by BNP levels [7]. Currently, BNP is advocated as an important indicator in determining hospital discharge and future prognosis [64]. Regarding the most efficacious monitoring frequency, Dhaliwal et al. [61] observed that multiple BNP measurements did not provide a prognostic advantage over a single measurement when considering “disease effect” or “disease modifiability.” However, as noted, these data were gathered from a relatively small population size (*n* = 203) given treatment independent of information gleaned from BNP levels, leaving the possibility that continuous monitoring still promises to provide additional indicators for treatment decisions [61].

## BNP monitoring

With the emergence of BNP as a useful biomarker, several techniques have been applied to assess its concentration in blood. What follows is a brief summary of assays, benchtop biosensors and implantable biosensors concluding with four areas we have identified as potential space for growth.

### Assays

The first assays for BNP were competitive immunoassay methods using radio-labeled tracers (RIAs: radio immunoassays) [61, 65]. However, these assays suffered from large differences in specificity due to the presence of several related peptides in plasma or tissues samples, which could interfere with a competitive immunoassay. To overcome these problems, a non-competitive immunoradiometric assay (IRMA) method based on a two-site sandwich immunometric measurement was proposed and has demonstrated increased specificity for BNP [66–68]. More recently, micromosaic immunoassays (μMIAs) have been developed to simultaneously screen for the presence of multiple cardiac biomarkers including myoglobin, cardiac Troponin I and C-reactive peptide. The μMIA represents both a step in the evolution in the immunosensor and a widespread sentiment for the necessity of monitoring cardiac biomarkers [69].

Though quickly acknowledged as a useful tool in monitoring HF, it took 12 years from its discovery until the first BNP assay received clearance from the Food and Drug Administration (FDA) in 2000. Table 1 shows the analytical characteristics of commercial BNP assays as stated by their respective manufacturers [70]. However, several studies demonstrate that there are marked differences in analytical performance and measured values among these commercial methods [44, 71–73].

**Table 1:** Analytical characteristics of commercial BNP assays as per the manufacturer (70)

Commercial diagnostic tests	Capture antibody	Detection antibody	Standard material	FDA cleared yes/no/claim
Abbott Architect, AxSYM iSTAT	NH_2_ terminus and part of the ring structure (Scios), murine monoclonal AB, aa 5–13	COOH terminus, murine monoclonal AB, aa 26–32	Synthetic BNP 32	Assist in diagnosis of HF Assess severity of disease
Alere^a^ Triage BNP	NH_2_ terminus and part of the ring structure (Scios), murine monoclonal AB, aa 5–13	BNP (Biosite), murine omniclonal AB, epitope not characterized	Recombinant BNP	Aid in diagnosis and severity assessment of HF Risk stratification of patients with ACS and HF FDA cleared
Beckman Coulter^a^ Access, Access 2, DxI	BNP (Biosite), murine omniclonal AB, epitope not characterized	NH_2_ terminus and part of the ring structure (Scios), murine monoclonal AB, aa 5–13	Recombinant BNP	Diagnosis HF Assess severity HF Risk ACS Risk HF
Siemens (Bayer) ACS 180, Advia Centaur, Advia Centaur CP	COOH terminus (BC-203) (Shionogi), murine monoclonal AB, aa 27–32	Ring structure (KY-hBNPII) (Shionogi), murine monoclonal AB	Synthetic BNP	Aid in diagnosis and severity assessment of HF Predict survival and likelihood of future HF in ACS patients
Siemens (Dade Behring) Dimension VISTA Dimension ExL	Ring structure (KY-hBNPII) murine monoclonal AB, aa 14–21	COOH terminus (BC-203), murine monoclonal AB, aa 27–32	Synthetic BNP 32	Aid in diagnosis and severity assessment of HF Predict survival and likelihood of future HF in ACS patients Pending FDA clearance
Shionogi	COOH terminus (BC-203), murine monoclonal AB, aa 27–32	Ring structure (KY-hBNPII), murine monoclonal AB	Synthetic BNP	Not FDA cleared
Tosoh ST AIA-PACK BNP	COOH terminus (BC-203), murine monoclonal AB, aa 27–32	Ring structure (KY-hBNPII), murine monoclonal AB	Synthetic BNP	Not FDA cleared

*CHF* congestive heart failure, *ACS* acute coronary syndrome, *aa* amino acid, *AB* antibody

^a^Both the Alere and Beckman systems use the same two antibodies, but due to their different assay formats, designation of the monoclonal and omniclonal antibodies as capture and detection antibody is not absolute

Of particular note is that commercially available BNP assays are heterogeneous and measure both pro-BNP and the breakdown products of BNP_1-32_ [73–75]. BNP_1-32_ varies according to disease progression being diagnostically significant, but thus far, we do not have the tools to accurately differentiate the molecular forms. So far, researchers are not capable of differentiating the molecular forms of these peptides, such as different cleavage points and post-translational modifications, which may vary depending on the pathophysiological state of chronic HF [72, 73, 76].

Though by and large assays have been effective in their specific realms, hours often pass from when a test is ordered until the results are ready. New developments have led to the production of point-of-care (POC) assays that allow for more rapid testing. Unfortunately, POC assays sacrifice reliability in pursuit of practicability as noted by [5, 77]. Variations in chip-based immunoassays such as that developed by Wang et al. [78] allow for faster assay time (15 min) and multiple marker quantification, but they still require trained technicians who, in order to provide necessary monitoring, must repeatedly withdraw blood samples for analysis, another drawback of many assay techniques [5]. In addition to these mechanical disadvantages, because of the temporal limitations of assays, monitoring BNP via assay is like trying to watch a film three or four frame per hour: You cannot see the continual motion of BNP levels. Therefore, in addition to being painful and time-consuming, assays suffer from the inability of capturing real-time information that reflects an individual’s cycle, direction and trends throughout the day [78, 79].

### Benchtop biosensors

Notwithstanding the availability of the above assays, biosensors continue to be developed. As stated, biosensors are electrical, optical, chemical or mechanical devices with the capability to detect biological species selectively [80, 81]. Put another way, a biosensor is simply an electronic device that produces electronic signals as the result of biological interactions [80]. Like assays, to be reliable, biosensors must possess a high level of sensitivity and selectivity for a desired molecule and are therefore usually designed to target one species. In addition, to be clinically useful, a biosensor must give rapid feedback. The economics of biosensors are such that defining a balance between the competing qualities of sensitivity, selectivity and response time is often hard to strike and becomes somewhat of an engineering tug-of-war as one must often take precedent at the expense of the other; for example, increasing selectivity with the addition of a zwitterionic semipermeable nanomembrane might also degrade response time [82]. Each biosensor, therefore, is developed with specific application in mind and therefore a specific equilibrium between sensitivity, selectivity and response time. More will be said of sensitivity and selectivity in section “Opportunities for future development” below.

There are many biosensor concepts. To use the classification set forth, they are generally divided into two groups: catalytic sensors and affinity sensors. Catalytic sensors employ enzymes, microorganisms or whole cells to catalyze biological interactions with a target substance. When this target substance is altered, it creates a change in the electrical properties of the device, thereby generating a response signal. Affinity systems use antibodies, receptors, nucleic acids or other members of a binding pair to form specific molecular interactions. When these reactions occur, there is a change in the molecular dipoles of the binding pair, which can be sensed by the device and therefore serve as a response signal.

Most similar to immunoassays are electrochemical enzyme immunoassays [82–84] and on-chip enzyme immunoassays such as the microfluidic device with a portable surface plasmon resonance (SPR)-based sensor reported by Kurita et al. [85]. This SPR-based sensor indirectly measure BNP by quantifying the activity of acetylcholine esterase attached to an anti-BNP antibody. Teramura et al. [86] have developed an SPR biosensor on a sandwich-type immunoassay using two kinds of monoclonal antibodies. A clever application of streptavidin-conjugated nanobeads reported by Mischak et al. [87] allows an SPR sensor to detect changes at the level of picogram (pg/ml), a concept with many potential applications to biosensors. Other concepts for detecting cardiac biomarkers have also been developed such as nanofluidic channel-based and nanoparticle-enhanced florescence-based sensors [86], a silicon nanosensor for diagnosis of cardiovascular proteomic markers [88] and a polyaniline nanowire-based conductometric biosensor [89].

Though clinically useful, in vitro biosensors suffer from many of the same disadvantages as assays. Because they are extra-dermal, body fluids must either be withdrawn for analysis or a probe must be temporarily inserted into tissue to measure internal conditions; both are time-consuming, inconvenient and painful. In addition, the scope of most biosensors is limited to in vitro analysis either because they are too large to be effectively implanted or because they contain toxic materials and therefore cannot be used in vivo, a complication that will become significant as we discuss implantable biosensors below.

### Implantable biosensors

To facilitate discussion, we will group implantable biosensors into two functional categories based on location: subcutaneous and vascular. As a class, implantable biosensors offer many advantages over external biosensors and assays, the greatest being valuable, real-time information. Real-time information allows more rapid modification of treatment to suit dynamic physiological conditions as well as earlier detection of threatening disease states [90]. As noted by Hirsch et al. [91], because traditional analyte monitors provide only a snapshot of physiological conditions with no indication to trend or rate of change, adjusting treatment and predicting disease states on this basis involves considerable guesswork, especially when self-administered as in the case of diabetes. Real-time monitoring permits moment-by-moment understanding of an individual’s biological rhythms throughout the day and how an individual responds to such things as exercise, meals, pregnancy or other events [7, 91].

Additional benefits of the implantable biosensor over assays or benchtop biosensors include the avoidance of much of the inconvenience, pain and time demands of drawing blood for periodic, quotidian or post-prandial analysis. There are, of course, psychological as well as economic advantages and should not be underestimated. In determining the causes of low adherence to monitoring schedules, recent evidence has pointed not to lack of patient education, as was previously thought, but to the consistent psychological and economic burden found with in vitro analysis [92, 93].

Because of their obvious application to diabetes management, much effort has been made to develop continuous glucose monitoring system (CGMS) [94–97]. Due to the molecular nature of glucose, the actual monitoring mechanisms of glucose, chemosensors, are technically different from the catalytic or affinity sensors used to quantify levels of proteins like BNP. The implantable glucose biosensors described below, therefore, serve to demonstrate not proof of technology, but proof of concept and of overall design.

#### Subcutaneous implantable biosensors

Subcutaneous biosensors are often needle-shaped and transdermal with the actual sensor implanted in the subcutaneous tissue and a lead wire extending to an extra-dermal monitoring display. Many implantable glucose sensors have been developed [91, 95, 97–99]. The first commercially glucose monitoring systems were initially labeled for up to 3 days of wear [100]. Next, the wear time was extended to 7 days [101]. Many sensors are coupled to insulin pumps that automatically regulate glucose levels within proscribed levels, alerting the patient when a threshold is breached [102, 103].

While subcutaneous sensors provide the benefits of real-time monitoring and do not require body fluids to be drawn for analysis, because they are part external, they possess disadvantages of decreased free movement, poor esthetic appeal and risk of infection [91, 104]. Extensive ongoing research is aiming to increase the sensor lifespan [105–109]. So far, patient trials have returned favorable results [110–112].

#### Vascular implantable biosensors

As suggested by the name, vascular implantable biosensors are inserted into a vessel and can either be catheter based or non-catheter based. Because of their suitability to cardiovascular applications, the majority of vascular implantable biosensors have been developed to be specific for cardiovascular biomarkers such as BNP.

Catheter-based systems can be deployed on an intracardiac lead or other delivery device as a stand-alone system or they can be incorporated onto an implantable medical device such as a pacemaker, defibrillator or cardiac resynchronization system [91]. Non-catheter-based sensors are often incorporated onto stents and are among the more advanced of this class [82]. Both are contained completely within the body (i.e., without any external anchors, leads or probes) and transmit transdermal signal to an external monitoring device via a telemetry device that utilizes radio waves [113, 114].

Because they are completely internal, implantable biosensors sidestep complications such as decreased free movement and infection which are common to transdermal catheters and needle-like probes [104, 115]. Non-catheter-based sensors have the added advantage of being localized to a specific intravascular region, thereby decreasing risk of damage to arterial walls.

As the next step in the biosensor evolution, vascular biosensors are still very much in the developmental process. While internalization is the implantable biosensor’s greatest asset, it is also its greatest obstacle as two complications quickly arise: evasion of the foreign body response and renewability; both are necessary to insure the longevity of the device. Therefore, in addition to the technical hurdles of selectivity, sensitivity and responsiveness present in other sensors, in vivo applications must also hurdle sustainability within a hostile host environment and continuity of function [110].

### Opportunities for future development

#### Foreign body response

One problem specifically pertinent to vascular implantable biosensors and yet to be overcome is the matter of the foreign body response, or biofouling. As defined, biofouling is the accumulation of proteins, cells and other biological materials on a surface. This accumulation causes loss in sensitivity and/or activates the immune response that encapsulates the sensor in fibrous scar tissue as part of the body’s normal healing process. Biofouling arises with any implantable device and therefore has been investigated by many scientists with some success [116, 117].

Gant et al. [118] have developed a coating that expands and contracts with variances in temperature, thus in essence sloughing off any buildup of cells while changing the surface hydrophobicity of the implant to deter further buildup. Though conceptually appealing, complexities arise when considering how to affect and control such a temperature change in vivo. Furthermore, due to the temperature sensitive nature of the hydrogel membrane, it is unclear what effect the bodies’ natural temperature rhythms (daily, menstrual, etc.) would have on the efficacy of the material which demands calibration within a specific temperature range.

Other attempted solutions to the foreign body response have been addition of an external silicon membrane, composite layers of carbon nanotubules and other nanoporous membranes [118], zwitterionic coatings [119], thermoresponsive double network nanocomposite (DNNC) membrane [120] and matrices of proteases designed to degrade proteins bound to biosensor surfaces [121]. Though preliminary studies on glucose sensors have been hopeful, because the exact mechanism of biofouling is still under investigation, attempts to prevent or deter the process are speculative at best [81].

#### Sensor renewability

Current catalytic and affinity-based biosensors are designed such that analysis of a particular molecule will eventually exhaust a finite supply of enzyme or molecular binding partner. Of course, in vitro, this presents little problem, because one can refresh the sensor by replacing, in the case of an immunosensor, the supply of antibodies [81]. In vivo, however, the story is something different as the device is supposed to embrace the concept of laissez-faire; e.g., the device needs to be self-sustaining for a significant amount of time. Concomitant with the exhaustion of antibodies is the production of waste, something, again, inconsequential in vitro, but unacceptable in vivo where toxicity quickly becomes an issue [82].

Again taking example of affinity-based sensors, if the uptake and release of antigen from the sensing surface can be controlled by external factors, the antigen–antibody interaction will, in essence, be reusable allowing this type of implantable sensors to find much wider application and acceptance. This concept of a reusable sensor has become known as a “cleanable” sensing surface. Application of switchable or smart surfaces might be applied to allow for such a cleanup.

Smart surfaces are those which are able to respond to environmental stimuli, thereby forming a type of switch given certain conditions [122]. Various switchable surfaces initiated by external factors, including photon [123, 124], charge [125–127], pH [128, 129] and temperature [130] have been extensively studied. Electrical potential stimulation is a widely used method for controlling surface properties. It has been employed by using low density self-assembly monolayer for the design of switchable hydrophobic/hydrophilic surfaces by reversible conformational transitions [131, 132]. Electrical potential stimulation could be applied to such an immunosensor allowing the stimulus to “clean” the sensor.

#### Sensitivity and selectivity

Briefly mentioned above, more must be said of the balance between sensitivity and selectivity. Sensitivity is the magnitude of a biosensor’s response to a particular analyte. Selectivity refers to its ability to respond only to a specific analyte among the plethora found in biofluids [129]. Sensitivity and selectivity depend on the materials used to build the sensor and on the design of the sensor itself and often represent competing interests as one often improves at the expense of the other.

Both represent significant challenges in the development and acceptance of biosensors. To be useful in early disease states, where biomarkers levels are often less than tens of picomoles, sensors must be highly sensitive and specific [81]. Though there is not yet a sensor developed for BNP that is highly sensitive and specific, nanotechnology presents many promising applications to implantable biosensors. Vaddiraju et al. [81] have provided a review of this emerging synergy. To showcase the technology, we will briefly discuss silicon nanowire (SiNW) clusters.

Chua et al. [133] have reported the development of SiNW cluster arrays. Based on electrical detection of antibody–analyte interaction, they have demonstrated sensitivity to cardiac troponin-T (cTnT) at 1 fg/mL in buffer solution and down to 30 fg/mL in undiluted human serum with a 3:1 signal-to-noise ratio. Given high levels of total protein concentration, this concept has also demonstrated high specificity. Because the sensor is electronics based, it can be queried every second, allowing real-time detection of a particular analyte. The sensor boasts a total of 36 clusters of 5 nanowires each, leaving the potential for monitoring of multiple analytes [133].

Perhaps the greatest drawback to the application of this nanotechnology to implantable biosensors is, ironically, its size. The sensor developed by Chua et al. [133] measures 40 mm × 25 mm at the base, hardly capable of in vivo implantation. Perhaps if the sensor were to be designed for single-analyte specificity (as opposed to >35 analytes), the sensor could be sufficiently miniaturized for in vivo application. Indeed, the larger, pluri-specific sensor could be used as a screening device where a smaller, unispecific device could be used to continuously monitor an analyte of interest. Another barrier to the widespread use of nanotechnology is its monetary cost. As the technology develops this cost will decrease, but in status quo, economic barriers inhibit nanotechnology’s development by researchers who cannot afford the machinery to develop the sensor and its pilot use by physicians who cannot afford to venture from the beaten and less expensive path of bedside assays and benchtop biosensors. Notwithstanding these disadvantages, nanotechnology still holds promising potential.

## Summary and roadmap for future

As BNP continues to increase in its diagnostic importance, there will be increased interest in its quantification. To be able to classify a patient’s condition, administer-guided treatment, and measure the success thereof while predicting future outcomes, however, is no small task. In this review, we have attempted to survey the current research and progress in both the field of BNP research and how BNP is currently and will 1 day be measured in the human body. Ideally, the community will develop a device that can continuously measure BNP in vivo for extended periods of time.
